# Comparison of Influence of Blood Pressure and Carotid-Femoral Pulse Wave Velocity on Target Organ Damage in Hypertension

**DOI:** 10.3389/fcvm.2022.934747

**Published:** 2022-07-05

**Authors:** Huijuan Chao, Yan He, Qian Wang, Yaya Bai, Alberto Avolio, Xueqin Deng, Junli Zuo

**Affiliations:** ^1^Department of Geriatrics, Ruijin Hospital, Shanghai Jiao Tong University School of Medicine, Shanghai, China; ^2^Department of Cardiovascular Medicine, Ruijin Hospital, Shanghai Jiao Tong University School of Medicine, Shanghai, China; ^3^Macquarie Medical School, Faculty of Medicine Health and Human Sciences, Macquarie University, Sydney, NSW, Australia

**Keywords:** carotid-femoral pulse wave velocity, arterial stiffness, left ventricular hypertrophy, carotid intima-media thickness, hypertension

## Abstract

**Objectives:**

Assessment of target organ damage (TOD) is an important part of the diagnosis and evaluation of hypertension. Carotid-femoral pulse wave velocity (cf-PWV) is considered to be the gold-standard for noninvasive arterial stiffness assessment. This study aims to analyze the risk of TOD in people with different phenotypes of peripheral blood pressure and cf-PWV.

**Methods:**

The study cohort was recruited from December 2017 to September 2021 at Ruijin Hospital in Shanghai. It was divided into 4 groups according to peripheral blood pressure (pBP) and cf-PWV. TOD was assessed as carotid intima-media thickness (CIMT), chronic kidney disease (CKD), urinary albumin-creatinine ratio (ACR), estimated glomerular filtration rate (eGFR) and left ventricular mass index (LVMI).

**Results:**

A total of 1,257 subjects (mean age 53.13 ± 12.65 years, 64.2% males) was recruited. Age, body mass index (BMI) and fasting blood glucose (FBG), as well as peripheral systolic blood pressure (pSBP), peripheral diastolic blood pressure (pDBP), peripheral pulse pressure (pPP) were significantly different in the four groups (*P* < 0.01). eGFR, ACR, LVMI and CIMT were significantly different among different groups (*P* < 0.01). The risk of ACR abnormality was significantly higher in the group with elevated pBP (*P* = 0.005, OR 2.264, 95%CI 1.277–4.016; and in the group with elevated pBP and cf-PWV (*P* = 0.003, OR 1.482, 95%CI 1.144–1.920), while left ventricular hypertrophy (LVH) was significantly higher in the group with elevated cf-PWV (*P* = 0.002, OR 1.868, 95%CI 1.249–2.793).

**Conclusion:**

Different profiles based on the status of PBP and cf-PWV associated with different TOD. Individuals with higher pBP have an increased risk of ACR abnormality, while individuals with only cf-PWV elevated have a higher risk of LVH.

## Introduction

The Chinese Hypertension Survey (2012–2015) found that the crude prevalence rate of hypertension among Chinese adults (≥18) was 27.9% (weighted rate was 23.2%). The prevalence of hypertension in young people (18–34 years old) is 5.2%, and that of those ≥75 years of age is 59.8%. It is estimated that the number of adult hypertensive patients over 18 years of age in China is 245 million (1). Hypertension is a lifelong disease. Long-term hypertension can cause damage of central organs such as heart, brain, and kidneys. Assessing whether there is target organ damage is an important part of the diagnosis and evaluation of hypertension (2). Subclinical target organ damage related to cardiovascular disease is also an important part of cardiovascular risk assessment (3).

The characteristic changes of atherosclerosis are thickening and inflammation of the arterial wall (4) and associated arterial stiffness. Carotid-femoral pulse wave velocity (cf-PWV) is used to measure arterial stiffness conveniently and non-invasively. cf-PWV is considered to be an early marker of cardiovascular risk in hypertension, chronic kidney disease (CKD) and in the general population (5–7). Cf-PWV also has a significant correlation with mortality in hypertension and CKD populations (5, 6) .

Studies conducted in different populations suggested that the specific correlation between blood pressure, cf-PWV and target organ damage (TOD) is still uncertain. For example, the Finn-Home study (8) suggested that cf-PWV was significantly increased in patients with occult and persistent hypertension, and the risk of TOD represented by Cornell voltage and carotid intima-medial thickness (cIMT) was also significantly increased. When considering systolic home blood pressure (BP) levels, the difference in TOD becomes insignificant. Another study (3) suggested that in untreated middle-aged hypertensive patients without diabetes, arterial stiffness assessed by cf-PWV had a low correlation with left ventricular hypertrophy (LVH) and microalbuminuria. cf-PWV is mainly related to age, low-density lipoprotein cholesterol level and pulse pressure. In the healthy population, age and BP are the main predictors of increased cf-PWV, LVH, intima-media thickness (IMT) and the presence of carotid plaque (7).

The aim of this study was to analyze the risk of TOD in people with different BP and cf-PWV. This is to promote patient management in an individualized manner, with precise diagnosis and treatment. This approach will avoid considering only a single marker (such as only considering BP or arterial stiffness), which can lead to being over-treated or under-treated.

## Methods

### Study and Population

A total of 1,257 participants (age ≥18 years) who received a routine physical examination at Ruijin Hospital, Shanghai, China, between December 2017 and September 2020 was recruited. Subjects with any history of occlusive arterial disease history such as myocardial infarction or acute coronary syndrome, transient ischemic attack or stroke were excluded from the study. 1,300 subjects were invited, of whom 1,257 were enrolled (43 lacked cf-PWV data). The protocol received Ruijin Hospital ethics approval and all participants provided written informed consent.

### Measurements

All patients had their medical history taken, underwent physical examination and collection of anthropometric data. Height was mea-sured with a wall-mounted stadiometer to the nearest 0.5 cm with-out shoes and body weight was measured on a balance calibrated to the nearest 0.1 kg with minimum clothing and without shoes. Measurements were made with an up-stretched tape meter and recorded to the nearest 0.1 cm. Body height and weight were measured without shoes. Anthropometric measures such as body mass index (BMI) were calculated using these anthropometric data. Status of current smoking was defined as having smoked the last cigarette within 1 week of when BP measurements were taken.

Blood samples were obtained after 12–14 h of overnight fasting. Serum Total Cholesterol (TC), Low-density Lipoprotein Cholesterol (LDL-C), High-density Lipoprotein Cholesterol (LDL-C), Triglycerides (TG), Fasting Blood Glucose (FBG), and serum Creatinine (Cr) were obtained from patient medical records. The Modification of Diet in Renal Disease (MDRD) formula was used to calculate the esti-mated Glomerular Filtration Rate (eGFR) (9). Urinary albumin and creatinine were measured from the urine sample.

Seated BP measurements were obtained in 12-h fasting individuals in the morning (7–9 am) with a standard manual brachial cuff sphygmomanometer after a 10-min rest and using the average of two readings in both arms. Pulse pressure (PP) was calculated as the difference between systolic BP (SBP) and diastolic BP (DBP) and mean arterial pressure (MAP) was calculated as (DBP) + (PP)/3. Measurement of cf-PWV was performed using the SphygmoCor CVMS system (AtCor Medical PtyLtd, Sydney, Australia) as per the manufacturer's protocol in a supine position. Radial artery pressure waveforms were recorded using the high-fidelity tonometer for at least 10 s, until a stable radial tonometric pressure trace was obtained. cf-PWV was calculated as the ratio of the direct distance between the carotid and femoral sites of measurements and pulse transit time calculated as a direct delay between the two waves in relation to the *r* wave of the electrocardiogram.

Carotid IMT (CIMT) was examined bilaterally using high-resolution echocardiography Doppler ultrasound (HD11EX Ultrasound; Philips Medical Systems, Andover, MA, USA) with a broad-band linear array transducer (multiple frequency: 4–12 MHz). IMT was measured on both the left and right common carotid artery starting ~1.5 cm proximal to the carotid artery bulb. Three recordings were taken, and the mean value was calculated for each side. “Plaque = 0” referred to the absence of plaque, while “plaque = 1” referred to the presence of plaque.

M-mode or two-dimensional echocardiography was used to assess the measurement of left ventricular end-diastolic diameter (LVEDd), interventricular septal diameter (IVSd), posterior wall thickness at end-diastole diameter (PWTd) and left ventricular end-systolic diameter (LVESd) from the parasternal view (10), and then left ventricular mass (LVM) was calculated through those parameters using related for-mulas. Left ventricular mass index (LVMI) was assessed by LVM divided by body surface area (BSA). LVM (g) = 0.8×{1.04× [(LVEDd + PWTd + IVSd)^3^ – (LVEDd)^3^]} +0.6 and LVMI (g/m^2^) = LVM/BSA (11). The abovementioned measurements were made according to the guidelines of the American Society of Echocardiography (ASE) (12).

### Definition of Hypertensive TOD

Asymptomatic hypertensive TOD include cardiac, arterial, and renal TOD. Left ventricular hyper-trophy (LVH) was defined as LVMI of 115 g/m^2^ (male) or LVMI of 95 g/m^2^ (female) (13). Renal TOD was defined as microalbuminuria (MAU) (UACR 30) and renal dysfunc-tion (RD) as creatinine clearance rate of 60 mL/min/1.73 m^2^. Urinary ACR was calculated using the formula: ACR (mg/g) = urinary albumin/urinary creatinine. eGFR < 60 ml/min per 1.73 m^2^ was defined as CKD and ACR >30 mg/g was defined as microalbuminuria. Arterial TOD was defined as increased CIMT (CIMT > 900 um) or as presence of arterial plaque.

The included subjects were grouped according to peripheral BP and cf-PWV. Group(control): normal BP and cf-PWV, with peripheral BP <140/90 mmHg and cf-PWV < 10 m/s; Group (PBP): only elevated BP, normal cf-PWV, with peripheral BP ≥ 140/90 mmHg and cf-PWV <10 m/s; Group(cf-PWV): Only with elevated cf-PWV with peripheral BP < 140/90 mmHg and cf-PWV ≥ 10 m/s. Group (PBP/cf-PWV): abnormal peripheral BP and cf-PWV; peripheral BP ≥ 140/90 mmHg and cf-PWV ≥ 10 m/s.

### Statistical Analysis

Continuous variables are expressed as mean ± SD, and frequencies (percentage) are reported for categorical variables. Continuous and categorical variables were compared using *t*-test and Chi-square test respectively for males and females. To compare continuous and categorical variables among the four groups, Analysis of Variance (ANOVA) and Chi-square test respectively were used. Logistic regression analysis was used to calculate the Odds Ratios (ORs) adjusted for cardiovascular risk factors including age, sex, BMI, smoking, hypertension, Antihypertensive drugs (yes or no), HDL, LDL, fasting blood glucose, peripheral mean arterial pressure, heart rate. Analyses were performed with SPSS 26.0 for Windows (SPSS Inc, Chicago, IL, USA). A two-sided *p* < 0.05 was considered statistically significant.

## Results

### Baseline Characteristics of Studied Population

A total of 1,300 subjects was enrolled in this study, 43 of which were excluded due to data abnormalities, and finally 1,257 subjects (mean age 53.13 ± 12.65 years, 64.2% males) were recruited in our study (Table 1). Results showed that males had a higher rate of smoking history, as well as higher levels of BMI and triglyceride (*P* < 0.01), while females had higher levels of total cholesterol, LDL and HDL (*P* < 0.01). There were no significant differences for peripheral SBP (pSBP) and heart rate (HR) between males and females. Males had a higher level of pDBP, while pPP was significantly higher in females. However, in terms of medical history, the prevalence of hypertension and the rate of antihypertensive treatment were significantly higher in men, as well as cf-PWV (*P* < 0.05). In the case of TOD, eGFR, ACR and LVMI were significantly higher in male than in female.

**Table 1:** Baseline characteristics of participants by sex.

	**Overall** ***N* = 1,257**	**Men** ***N* = 807**	**Women** ***N* = 450**	***P* Value**
**Cardiovascular risk factors**
Age, years	53.13 ± 12.65	52.05 ± 12.62	55.00 ± 12.78	<0.01
Smoking history, *n* (%)	215 (17.1)	199 (24.24)	16 (3.47)	<0.01
Body mass index, kg/m^2^	25.32 ± 3.90	25.96 ± 3.82	24.29 ± 3.98	<0.01
Fasting plasmaglucose, mmol/l	5.82 ± 1.81	5.9 ± 1.78	5.70 ± 1.86	0.068
Total triglycerides, mmol/L	1.91 ± 1.56	2.07 ± 1.76	1.62 ± 1.05	<0.01
Total cholesterol, mmol/l	4.79 ± 1.07	4.68 ± 1.08	4.95 ± 1.01	<0.01
High-density lipoprotein, mmol/l	1.15 ± 0.34	1.08 ± 0.35	1.26 ± 0.29	<0.01
Low density lipoprotein, mmol/l	3.12 ± 0.81	3.06 ± 0.81	3.20 ± 0.78	0.006
**Peripheral blood pressure**				
Peripheral systolic blood pressure, mmHg	130.65 ± 18.56	131.45 ± 17.38	129.43 ± 20.60	0.075
Peripheral diastolic pressure, mmHg	76.69 ± 11.96	78.12 ± 11.52	74.19 ± 12.34	**0.019**
Heart rate bpm	69.25 ± 10.39	69.28 ± 10.26	69.38 ± 10.95	0.865
Peripheral pulse pressure,mmHg	53.96 ± 13.33	53.33 ± 12.50	55.24 ± 14.67	**0.019**
**Asymptomatic hypertension impaired target organs**
Creatinine clearance rate, mL/min 1.73 m^2^	90.07 ± 16.98	92.06 ± 17.70	86.49 ± 15.32	<0.01
Urinary creatinine ratio rate, mg/mmol	6.43 ± 28.72	7.78 ± 34.11	3.45 ± 4.57	0.008
Left ventricular mass index, g/m^2^	104.05 ± 26.35	107.77 ± 26.52	96.18 ± 24.72	<0.01
Carotid intima-media thickness, mm	0.74 ± 0.14	0.75 ± 0.15	0.72 ± 0.12	0.061
cf-PWV m/s	8.24 ± 2.02	8.35 ± 1.96	8.06 ± 2.11	0.015
**Prevalence of target organ damage**				
eGFR <60 ml/min per 1.73 m^2^, *n* (%)	34 (3.1)	17 (2.4)	17 (4.4)	0.075
Urinary creatinine ratio rate >30 mg/g, *n* (%)	117 (21.6)	79 (20.9)	38 (23.3)	0.368
Left ventricular hyper-trophy, *n* (%)	260 (35.8)	154 (30.8)	106 (20.6)	<0.01
Arterial target organ damage, *n* (%)	348 (50.4)	241 (52.9)	107 (45.7)	0.07
cf-PWV≥10m/s, *n* (%)	205 (16.3)	133 (16.5)	72 (16.0)	0.825
**Clinical disease and treatment**
Hypertension, *n* (%)	401 (31.9)	294 (36.4)	107 (23.8)	<0.01
Antihypertensive treatment, *n* (%)	382 (30.4)	282 (34.9)	100 (100)	<0.01
Diabetes treatment, *n* (%)	176 (13.7)	120 (14.6)	56 (12.1)	0.218

*Data are means and standard deviation or numbers with percentages in parentheses. Student t test and chi-squared test were conducted to compare the differences between men and women for quantitative and qualitative variables, respectively; cf-PWV, carotid femoral pulse wave velocity*.

After the cohort was grouped according to BP and cf-PWV, there were statistically significant differences in age, BMI, blood glucose, LDL, pSBP, pDBP, pPP, HR, eGFR, ACR, LVMI, CIMT, prevalence of hypertension and antihypertensive treatment among the four groups(*P* < 0.01). There was no statistical difference in hypoglycemic treatment (Table 2).

**Table 2:** Demographic characteristics of groups based on peripheral blood pressure and cf-PWV.

	**Overall**	**Group (control)**	**Group (PBP)**	**Group (cf-PWV)**	**Group (PBP/cf-PWV)**	***P* Value**
	***N* = 1,257**	***N* = 777**	***N* = 275**	***N* = 87**	***N* = 118**	
**Cardiovascular risk factors**						
Age, y	53.13 ± 12.65	50.75 ± 11.77	51.79 ± 11.69	66.00 ± 11.32	62.42 ± 11.52	<0.01
Sex (male, %)	807 (64.20)	494 (63.58)	180 (65.45)	59 (67.82)	74 (62.71)	0.819
Smoker, *n* (%)	215 (17.1)	137 (17.63)	47 (17.09)	13 (14.94)	18 (15.28)	0.871
Body mass index, kg/m^2^	25.32 ± 3.90	24.88 ± 3.73	26.46 ± 4.16	24.83 ± 3.29	25.94 ± 4.18	<0.01
Fasting plasma glucose, mmol/l	5.82 ± 1.81	5.61 ± 1.46	5.92 ± 2.01	6.52 ± 2.61	6.47 ± 2.30	<0.01
Total triglycerides, mmol/L	1.91 ± 1.56	1.83 ± 1.32	2.13 ± 2.23	1.76 ± 1.26	2.00 ± 1.20	0.061
Total cholesterol, mmol/l	4.79 ± 1.07	4.83 ± 1.02	4.78 ± 1.22	4.47 ± 0.95	4.79 ± 1.07	0.057
High-density lipoprotein, mmol/l	1.15 ± 0.34	1.17 ± 0.35	1.09 ± 0.25	1.17 ± 0.51	1.10 ± 0.24	0.08
Low density lipoprotein, mmol/l	3.12 ± 0.81	3.16 ± 0.80	3.08 ± 0.83	2.89 ± 0.75	3.11 ± 0.82	0.043
**Peripheral blood pressure**						
Peripheral systolic blood pressure, mmHg	130.65 ± 18.56	120.04 ± 11.72	150.61 ± 10.20	128.91 ± 8.32	155.32 ± 13.82	<0.01
Peripheral diastolic pressure, mmHg	76.69 ± 11.96	72.07 ± 9.32	87.30 ± 10.77	72.55 ± 9.08	85.47 ± 11.43	<0.01
Heart rate bpm	69.25 ± 10.39	68.67 ± 10.23	69.14 ± 10.95	70.55 ± 9.25	72.42 ± 10.36	0.002
Peripheral pulse pressure,mmHg	53.96 ± 13.33	47.97 ± 8.83	63.31 ± 12.79	56.36 ± 9.92	69.85 ± 15.24	<0.01
**Asymptomatic hypertension impaired target organs**						
Creatinine clearance rate, mL/min 1.73 m^2^	90.07 ± 16.98	91.56 ± 15.65	90.57 ± 16.53	87.36 ± 18.30	81.93 ± 21.76	<0.01
Urinary creatinine ratio rate, mg/mmol	6.43 ± 28.72	3.91 ± 15.54	8.72 ± 33.28	21.27 ± 75.38	8.6 ± 19.80	0.001
Left ventricular mass index, g/m^2^	104.05 ± 26.35	98.28 ± 22.58	111.82 ± 28.54	114.98 ± 30.36	116.92 ± 29.88	<0.01
Carotid intima-media thickness, mm	0.74 ± 0.14	0.72 ± 0.12	0.74 ± 0.13	0.79 ± 0.17	0.81 ± 0.18	<0.01
cf-PWV m/s	8.24 ± 2.02	7.29 ± 1.18	8.28 ± 1.01	11.54 ± 1.62	11.99 ± 1.45	<0.01
**Prevalence of target organ damage**						
eGFR <60 ml/min per 1.73 m^2^, *n* (%)	34 (3.1)	12 (1.9)	7 (12.8)	3 (3.8)	12 (11.1)	0.026
Urinary creatinine ratio rate >30 mg/g, *n* (%)	117 (21.6)	57 (13.1)	34 (27.6)	9 (18.4)	17 (32.6)	<0.01
Left ventricular hyper-trophy, *n* (%)	260 (35.8)	124 (27.4)	65 (44.8)	36 (60.0)	35 (50.7)	<0.01
Arterial target organ damage, *n* (%)	348 (50.4)	181 (43.7)	70 (47.9)	43 (76.8)	54 (73.0)	<0.01
**Clinical disease and treatment**						
Hypertension, *n* (%)	401 (31.9)	216 (28.3)	104 (36.1)	35 (40.2)	46 (38.7)	0.007
Antihypertensive treatment	382 (30.4)	214 (27.4)	92 (33.7)	30 (34.5)	46 (38.7)	0.026
Diabetes treatment	173 (13.8)	105 (13.5)	33 (12.0)	15 (17.2)	20 (16.9)	0.448

*Values are mean ± SD for continuous variables or n (%), analysis of variance for numeric variables and chi-square test for categoric variables*.

*Groups by peripheral blood pressure and cf-PWV*.

*Group(control): normal blood pressure and cf-PWV group, with peripheral blood pressure less than 140/90mmHg and cf-PWV < 10 m/s*.

*Group (PBP): only elevated blood pressure group, normal cf-PWV group, with peripheral blood pressure ≥140/90 mmHg and cf-PWV < 10 m/s*.

*Group(cf-PWV): only the group with elevated cf-PWV had peripheral blood pressure < 140/90 mmHg and cf-PWV ≥ 10 m/s*.

*Group (PBP/cf-PWV): the group with abnormal peripheral blood pressure and cf-PWV, peripheral blood pressure ≥140/90 mmHg and cf-PWV ≥ 10 m/s*.

### Correlation Between Asymptomatic Hypertensive TOD and Cardiovascular Risk Factors in Hypertension

EGFR was negatively correlated with pSBP (*P* < 0.01, *r* = −0.106) and pPP (*P* < 0.01, *r* = −0.141). ACR was positively correlated with pSBP (*P* < 0.017, *r* = 0.092). lgACR was positively correlated with pSBP (*P* < 0.01, *r* = 0.210), pDBP (*P* < 0.01, *r* = 0.177), pMAP (*P* = 0.001, *r* = 0.133) and pPP (*P* = 0.001, *r* = 0.122). LVMI was positively correlated with pSBP (*P* < 0.01, *r* = 0.283), pDBP (*P* < 0.01, *r* = 0.167), pMAP (*P* < 0.01, *r* = 0.513) and pPP (*P* < 0.01, *r* = 0.246). CIMT was positively correlated with pSBP (*P* < 0.001, *r* = 0.189), pMAP (*P* < 0.01, *r* = 0.156) and pPP (*P* < 0.01, *r* = 0.249). cf-PWV was negatively correlated with eGFR (*P* < 0.01, *r* = −0.230), and positively correlated with ACR (*P* = 0.004, *r* = 0.111), lgACR (*P* < 0.01, *r* = 0.174), LVMI (*P* < 0.01, *r* = 0.310) and CIMT (*P* < 0.01, *r* = 0.292) (Table 3).

**Table 3:** Correlation between asymptomatic hypertensive target organ damage and cardiovascular risk factors in hypertension.

**Variance**	**eGFR**		**ACR**		**lgACR**		**LVMI**		**CIMT**		**cf-PWV**	
	***r***	***P* value**	***r***	***P* value**	***r***	***P* value**	***r***	***P* value**	***r***	***P value***	***r***	***P* value**
Age, y	−0.372**	<0.01	−0.027	0.487	0.003	0.938	0.250**	<0.01	0.386**	<0.01	0.489**	<0.01
Sex (male = 1, female = 2)	−0.156**	<0.01	−0.07	0.07	−0.058	0.134	−0.203**	<0.01	−0.071	0.061	−0.068*	0.015
Body mass index, kg/m^2^	0.02	0.499	0.018	0.639	0.047	0.225	0.159**	<0.01	0.012	0.756	0.136**	<0.01
Hypertension	−0.002	0.937	−0.003	0.937	0.051	0.191	0.128**	<0.01	0.029	0.442	0.125**	<0.01
Antihypertensive treatment	−0.004	0.89	0.005	0.902	0.082*	0.034	0.134**	<0.01	0.047	0.214	0.133**	<0.01
Fasting plasma glucose, mmol/l	0.01	0.753	0.032	0.421	0.111**	0.005	0.090*	0.016	0.134**	<0.01	0.239**	<0.01
Diabetes treatment	0.000	0.996	0.094*	0.015	0.047	0.225	0.012	0.747	−0.035	0.352	0.05	0.075
Total triglycerides, mmol/L	−0.014	0.648	0.079*	0.044	0.133**	0.001	0.043	0.245	−0.021	0.588	0.107**	<0.01
Total cholesterol, mmol/l	−0.061*	0.046	0.065	0.098	0.093*	0.018	−0.092*	0.014	−0.082*	0.034	−0.045	0.142
High-density lipoprotein, mmol/l	−0.001	0.986	−0.039	0.322	−0.072	0.068	−0.114**	0.002	−0.095*	0.014	−0.117**	<0.01
Low-density lipoprotein, mmol/l	−0.073*	0.018	0.058	0.139	0.088*	0.025	−0.076*	0.041	−0.065	0.09	−0.05	0.112
Peripheral systolic blood pressure, mmHg	−0.106**	<0.01	0.092*	0.017	0.210**	<0.01	0.283**	<0.01	0.189**	<0.01	0.499**	<0.01
Peripheral diastolic pressure, mmHg	−0.008	0.785	0.075	0.051	0.177**	<0.01	0.167**	<0.01	0.01	0.787	0.276**	<0.01
Peripheral mean pressure, mmHg	−0.01	0.748	0.077	0.056	0.133**	0.001	0.513**	<0.01	0.156**	<0.01	0.250**	<0.01
Peripheral pulse pressure, mmHg	−0.141**	0.000	0.056	0.149	0.122**	0.002	0.246**	<0.01	0.249**	<0.01	0.446**	<0.01
Heart rate bpm	0.101**	0.001	0.115**	0.003	0.098*	0.011	−0.103**	0.005	−0.064	0.093	0.098**	0.001
cf-PWV m/s	−0.230**	<0.01	0.111**	0.004	0.174**	<0.01	0.310**	<0.01	0.292**	<0.01		1

^**^Correlation is significant at the 0.01 level (2-tailed); ^*^Correlation is significant at the 0.05 level (2-tailed).

### Multiple Stepwise Logistic Regression Analysis of Target Organ Damage in Four Group

According to multiple stepwise logistic regression analysis, regarding Group(control) as the reference group, Group(PBP) and Group(PBP/cf-PWV) had higher risks of ACR abnormality (*P* = 0.005, OR 2.264, 95%CI 1.277–4.016; *p* = 0.003, OR 1.482, 95%CI 1.144–1.920), while the risk of LVH was significantly increased in Group(cf-PWV) (*P* = 0.002, OR 1.868, 95%CI 1.249–2.793) after adjusting age, sex, BMI, smoking history, hypertension, antihypertensive drugs (yes or no), HDL, LDL, fasting blood glucose, pMAP, HR(beats per minute, bpm) (Table 4).

**Table 4:** Multiple factor binary logistic regression analysis of target organ damage in four group.

	**Categories**	***n* (%)**	**OR (95% CI)**	***P* value**
ACR	Group (control)	52 (11.8%)	Ref.	
	Group (PBP)	35 (29.7%)	2.264 (1.277–4.016)	0.005
	Group (cf-PWV)	10 (20.4%)	1.181 (0.727–1.919)	0.502
	Group (PBP/cf-PWV)	20 (38.5%)	1.482 (1.144–1.92)	0.003
LVMI	Group (control)	124 (27.1%)	Ref.	
	Group (PBP)	65 (46.1%)	1.208 (0.712–2.051)	0.483
	Group (cf-PWV)	36 (60.0%)	1.868 (1.249–2.794)	0.002
	Group (PBP/cf-PWV)	35 (50.7%)	0.866 (0.68–1.101)	0.240
eGFR	Group (control)	12 (1.8%)	Ref.	
	Group (PBP)	7 (2.9%)	0.999 (0.279–3.582)	0.999
	Group (cf-PWV)	3 (3.8%)	0.497 (0.158–1.558)	0.230
	Group (PBP/cf-PWV)	12 (11.1%)	1.275 (0.869–1.872)	0.214

*All variables adjusted for age, sex, body mass index, smoking, hypertension, Antihypertensive drugs (yes or no), high density lipoprotein, low density lipoprotein, fasting blood glucose, peripheral mean arterial pressure, heart rate(bpm/min)*.

*Groups by peripheral blood pressure and cf-PWV*.

*Group(control): normal blood pressure and cf-PWV group, with peripheral blood pressure <140/90 mmHg and cf-PWV < 10 m/s*.

*Group (PBP): only elevated blood pressure group, normal cf-PWV group, with peripheral blood pressure ≥140/90 mmHg and cf-PWV < 10 m/s*.

*Group(cf-PWV): only the group with elevated cf-PWV had peripheral blood pressure < 140/90 mmHg and cf-PWV ≥ 10m/s*.

*Group (PBP/cf-PWV): the group with abnormal peripheral blood pressure and cf-PWV, peripheral blood pressure ≥140/90 mmHg and cf-PWV ≥10 m/s*.

## Discussion

The main finding of our study is that the increase in cf-PWV or increase in peripheral SBP alone, have different effect on specific target organs. Elevated peripheral SBP was associated with an increase of ACR, depending on whether cf-PWV was increased, the OR was 2.264 and 1.482, respectively. When peripheral SBP increased, regardless of the cf-PWV level, all participants had a certain risk of kidney and heart damage. Elevated cf-PWV alone was likely to cause LVH (OR 1.868).

Previous studies suggested that central and peripheral BP are correlated with urinary albumin excretion and ACR (14, 15), suggesting that BP has a clear predictive effect on kidney damage. Our study suggested that hypertension participants with elevated cf-PWV have a lower risk of elevated ACR than participants with normal cf-PWV. We have the following inferences about this finding. The proportion of participants in Group (PBP/cf-PWV) receiving hypoglycemic and antihypertensive therapy was higher, and some antihypertensive drugs such as angiotensin receptor blockers or angiotensin converting enzyme inhibitors may have certain effects on renal function and urine protein levels (14). Compared with ACR, eGFR is a more stable index of renal function (16). Therefore, when the SBP and cf-PWV both increased, both eGFR and ACR showed a worsening trend. For some participants (with normal cf-PWV and elevated blood pressure), only the trend of worsening ACR was shown, and the more stable eGFR remained at a higher level.

A Chinese study (17) suggested that cf-PWV was significantly associated with CKD and microalbuminuria, but our study did not show the correlation between cf-PWV and renal function. We believe this could be related to participants' age. Another Chinese study included the elderly population, showing an increase in cf-PWV in this population (18). However, our study included the young and middle-aged physical examination population, and the quadratic non-linear model between age and cf-PWV may cause the difference in correlation with renal function impairment. In addition, part of the population in our study received hypoglycemic and antihypertensive therapy, but not all, which may also confound the results of the study.

Our study also showed that cf-PWV is more closely related to LVH, but no correlation between increased blood pressure and increased risk of LVH has been observed. Theoretically, aortic stiffness increases the effects of wave reflection of the pressure waveform, leading to various unfavorable hemodynamic consequences, such as increased left ventricular afterload, which can lead to damage of the heart muscle (17), and this is consistent with the relationship between cf-PWV and LVH. The lack of correlation between BP and heart damage may be related to the following factors: Our previous study (19) suggested that both peripheral pulse pressure (PPP) and central pulse pressure (CPP) were related to target organ damage in populations with hypertension. Compared with PPP, CPP has a higher predictive ability for target organ damage over 60 years old. A Chinese study (17) suggested that pulse pressure amplification (PPA, the difference between central and peripheral PP in the elderly was tightly associated with LVH.

BP elevation is generally the most important risk factor resulting in increased Left ventricular mass (LVM) and leading to Left ventricular hypertrophy (LVH). PWV were greatly dependent on age and blood pressure. A recent study by Yun M et al. confirmed that elevated BP precedes the development of LVH and hemodynamic properties are responsible for the development of cardiac enlargement, during the young-to-midlife adult age period (20). In our study, subjects in Group (cf-PWV) were older and had lower systolic blood pressure than Group (PBP/cf-PWV), perhaps older age could explain to our results. Another study in the elderly (average age 60) suggested that participants with a high CPP and high cf-PWV experienced significantly increased CVD risk. PP-arterial stiffness mismatch is common in the community. cf-PWV may modify the association of CPP with CVD risk, with greatest risk being observed in those with elevated CPP and cf-PWV (21). The impact of CPP, PPP, PPA on the risk of target organ damage in people of different ages needs to be further studied. But in any case, the amplification of PP is complex, may be affected by many factors, and it also affects the correlation between peripheral BP and heart damage. We will also analyze this issue in a future study. In this study, with increase in cf-PWV, the normal peripheral SBP group showed a higher risk of LVH than the elevated SBP group, suggesting that increased cf-PWV caused greater damage to the target organ. Even if antihypertensive drugs are used to reduce BP to the normal range, elevated cf-PWV still causes the same damage. This suggests that particular attention should be given to these patients in clinical intervention and follow-up.

Carotid IMT formation is a valid measure of subclinical TOD which is related to future cardiovascular events. IMT was evaluated with manual measurement while echo-tracking semi-automated system exist (22), they were not available for our study and so IMT was evaluated with manual measurement. We acknowledge that this may have contributed to some lack of data. Another possible explanation for the results is that the study subjects who underwent physical examinations, not all of subjects had hypertension, diabetes, etc. A clinical study by Maloberti et al. indicated that there was no difference in the increase of IMT or carotid plaque in the normal group with high blood pressure, and the odds of having arterial stiffness or carotid hypertension mediated organ damage in the high-normal group was not different to the normal group in multivariate analysis (23). Further studies are needed in individuals with hypertension alone.

At present, the correlation between BP, cf-PWV and TOD remains to be further studied. The African-PREDICT study (24) suggested that for a healthy young population, the correlation between CIMT, ACR and BP is weak, while the correlation between central retinal arteriolar equivalent, LVMI cf-PWV and BP is strong. Compared with central BP or ambulatory BP, clinical brachial artery BP had a stronger correlation with early TOD. In a sample of the African population, the auscultation BP recorded by a nurse during a single visit was independent and positively correlated with LVMI, cf-PWV, and log ACR (25). There is also study (26) suggesting that compared with 24-h peripheral BP, the correlation between 24-h central BP and hypertensive TOD is not better.

The heterogeneity of these research conclusions may be related to many factors including age, personal characteristics, comorbid diseases, race, treatment, PP amplification and other factors. The results of our study showed that antihypertensive therapy is related to lgACR, LVMI, and cf-PWV, and hypoglycemic therapy is related to ACR. Age, gender, and peripheral SBP were independent risk factors for LVMI. Blood glucose, triglycerides, cholesterol, low-density lipoprotein, peripheral SBP, and heart rate were independent risk factors for cf-PWV. This also suggested the joint effect of multiple risk factors in target organ damage and the mutual influence of these risk factors.

## Limitations

There are some shortcomings and limitations in this study. This is a cross-sectional study with a small sample size and large differences among the four groups of grouped samples, which may lead to bias in the results. At the same time, due to the small sample size, this study did not further conduct male and female grouping and age stratification analysis. There was a large proportion of people taking antihypertensive drugs in the study, the time on antihypertensive and different types of antihypertensive drugs may have differing effects on the target organs of hypertension. The cuff sphygmomanometer is cylindrical rather than conical, which may be more appropriate for obese participants with large upper arms.

## Conclusion

In conclusion, we found that BP and cf-PWV have different effects on TOD. This suggest that TOD is affected by many factors, and it is not comprehensive to consider only BP or PWV. Factors such as age, comorbid diseases, treatment plan should be considered. For example, studies have suggested that for untreated non-diabetic hypertensive patients, there was a correlation between proteinuria, cf-PWV and LVMI, suggesting that TOD was progressing in parallel (27). This can lead to the question whether this conclusion be extended to treated non-diabetic hypertensive patients or treated diabetic patients with hypertension, or whether it is suitable for patients of different ages. Further research is required to uncover the multiple factors associate with TOD in patients with hypertension.

## Data Availability Statement

The original contributions presented in the study are included in the article/supplementary material, further inquiries can be directed to the corresponding author/s.

## Ethics Statement

The studies involving human participants were reviewed and approved by Ruijin Hospital. The patients/participants provided their written informed consent to participate in this study.

## Author Contributions

HC, YH, and JZ designed the research study, wrote the manuscript, analyzed the data, drafted, and revised the manuscript. XD, QW, and YB performed the research. YH, JZ, and XD analyzed and interpreted data. JZ and AA contributed to the thorough reading of the manuscript and several revisions. All authors contributed to the article and approved the submitted version.

## Funding

This project was supported by the National Natural Science Foundation of China (Grant No. 81500190), Clinical Science and Shanghai Municipal Hospital New Frontier Technology Joint Project (SHDC12019X20), and Shanghai Municipal Commission of Health and Family Planning (Grant No. 2020YJZX0124).

## Conflict of Interest

The authors declare that the research was conducted in the absence of any commercial or financial relationships that could be construed as a potential conflict of interest.

## Publisher's Note

All claims expressed in this article are solely those of the authors and do not necessarily represent those of their affiliated organizations, or those of the publisher, the editors and the reviewers. Any product that may be evaluated in this article, or claim that may be made by its manufacturer, is not guaranteed or endorsed by the publisher.

## References

[1] Hu S. Report on cardiovascular health and diseases in China 2019: updated summary. Chin Circ J. (2020) 35:833–54. 10.3969/j.issn.1000-3614.2020.09.00134773920

[2] Joint Committee for Guideline r 2018. Chinese guidelines for prevention and treatment of hypertension-a report of the revision committee of Chinese guidelines for prevention and treatment of hypertension. J Geriatr Cardiol. (2019) 16:182–241. 10.11909/j.issn.1671-5411.2019.03.014 31080465 PMC6500570

[3] Rodilla E Costa JA Perez-Lahiguera F Gonzalez C Pascual JM. Relationship between increased arterial stiffness and other markers of target organ damage. Med Clin (Barc). (2010) 134:528–33. 10.1016/j.medcli.2009.09.042 20022065

[4] Blankenhorn DH Kramsch DM. Reversal of atherosis and sclerosis. the two components of atherosclerosis. Circulation. (1989) 79:1–7. 10.1161/01.CIR.79.1.1 2642753

[5] Blacher J Safar ME Guerin AP Pannier B Marchais SJ London GM. Aortic pulse wave velocity index and mortality in end-stage renal disease. Kidney Int. (2003) 63:1852–60. 10.1046/j.1523-1755.2003.00932.x 12675863

[6] Laurent S Boutouyrie P Asmar R Gautier I Laloux B Guize L. Aortic stiffness is an independent predictor of all-cause and cardiovascular mortality in hypertensive patients. Hypertension. (2001) 37:1236–41. 10.1161/01.HYP.37.5.1236 11358934

[7] Willum-Hansen T Staessen JA Torp-Pedersen C Rasmussen S Thijs L Ibsen H. Prognostic value of aortic pulse wave velocity as index of arterial stiffness in the general population. Circulation. (2006) 113:664–70. 10.1161/CIRCULATIONAHA.105.579342 16461839

[8] Hanninen MR Niiranen TJ Puukka PJ Kesaniemi YA Kahonen M Jula AM. Target organ damage and masked hypertension in the general population: the finn-home study. J Hypertens. (2013) 31:1136–43. 10.1097/HJH.0b013e32835fa5dc 23466942

[9] Greene T Bourgoignie JJ Habwe V Kusek JW Snetselaar LG Soucie JM. Baseline characteristics in the modification of diet in renal disease study. J Am Soc Nephrol. (1993) 4:1221–36. 10.1681/ASN.V451221 8305650

[10] Lang RM Bierig M Devereux RB Flachskampf FA Foster E Pellikka PA. Recommendations for chamber quantification: a report from the american society of echocardiography's guidelines and standards committee and the chamber quantification writing group, developed in conjunction with the european association of echocardiography, a branch of the european society of cardiology. J Am Soc Echocardiogr. (2005) 18:1440–63. 10.1016/j.echo.2005.10.005 16376782

[11] Devereux RB Alonso DR Lutas EM Gottlieb GJ Campo E Sachs I. Echocardiographic assessment of left ventricular hypertrophy: comparison to necropsy findings. Am J Cardiol. (1986) 57:450–8. 10.1016/0002-9149(86)90771-X 2936235

[12] Quinones MA Otto CM Stoddard M Waggoner A Zoghbi WA. Doppler Quantification Task Force of the N, et al. Recommendations for quantification of doppler echocardiography: a report from the doppler quantification task force of the nomenclature and standards committee of the american society of echocardiography. J Am Soc Echocardiogr. (2002) 15:167–84. 10.1067/mje.2002.120202 11836492

[13] Marwick TH Gillebert TC Aurigemma G Chirinos J Derumeaux G Galderisi M. Recommendations on the use of echocardiography in adult hypertension: a report from the european association of cardiovascular imaging (eacvi) and the american society of echocardiography (Ase). J Am Soc Echocardiogr. (2015) 28:727–54. 10.1016/j.echo.2015.05.002 26140936

[14] Oliveras A Garcia-Ortiz L Segura J Banegas JR Martell-Claros N Vigil L. Association between urinary albumin excretion and both central and peripheral blood pressure in subjects with insulin resistance. J Hypertens. (2013) 31:103–8. 10.1097/HJH.0b013e32835ac7b5 23137953

[15] Neisius U Bilo G Taurino C McClure JD Schneider MP Kawecka-Jaszcz K. Association of central and peripheral pulse pressure with intermediate cardiovascular phenotypes. J Hypertens. (2012) 30:67–74. 10.1097/HJH.0b013e32834e12d8 22134387

[16] Waikar SS Rebholz CM Zheng Z Hurwitz S Hsu CY Feldman HI. Biological variability of estimated gfr and albuminuria in Ckd. Am J Kidney Dis. (2018) 72:538–46. 10.1053/j.ajkd.2018.04.023 30031564 PMC6469385

[17] Bai B Teliewubai J Lu Y Yu S Xiong J Chi C. Comparison of pulse wave velocity and pulse pressure amplification in association with target organ damage in community-dwelling elderly: the northern shanghai study. Hypertens Res Offic J Japanese Soc Hypertens. (2018) 41:372–81. 10.1038/s41440-018-0027-3 29535455

[18] Lu Y Zhu M Bai B Chi C Yu S Teliewubai J. Comparison of carotid-femoral and brachial-ankle pulse-wave velocity in association with target organ damage in the community-dwelling elderly Chinese: the Northern Shanghai study. J Am Heart Assoc. (2017) 6:168. 10.1161/JAHA.116.004168 28219916 PMC5523744

[19] Zuo J Chu S Tan I Butlin M Zhao J Avolio A. Association of haemodynamic indices of central and peripheral pressure with subclinical target organ damage. Pulse. (2018) 5:133–43. 10.1159/000484441 29761089 PMC5939803

[20] Yun M Li S Yan Y Sun D Guo Y Fernandez C. Blood pressure and left ventricular geometric changes: a directionality analysis. Pulse. (2021) 78:1259–66. 10.1161/HYPERTENSIONAHA.121.18035 34455810 PMC8516684

[21] Niiranen TJ Kalesan B Mitchell GF Vasan RS. Relative contributions of pulse pressure and arterial stiffness to cardiovascular disease. Hypertension. (2019) 73:712–17. 10.1161/HYPERTENSIONAHA.118.12289 30661478 PMC6374179

[22] Maloberti A Meani P Varrenti M Giupponi L Stucchi M Vallerio P. Structural and functional abnormalities of carotid artery and their relation with eva phenomenon. High Blood Press Cardiovascul Prevent Offic J Ital Soc Hypertens. (2015) 22:373–9. 10.1007/s40292-015-0100-9 25986075

[23] Maloberti A Rebora P Occhino G Alloni M Musca F Belli O. Prevalence of hypertension mediated organ damage in subjects with high-normal blood pressure without known hypertension as well as cardiovascular and kidney disease. J Hum Hypertens. (2021) 21:6. 10.1038/s41371-021-00604-6 34493835

[24] Botha D Breet Y Schutte AE. Comparing the associations of clinic vs. ambulatory blood pressure with subclinical organ damage in young healthy adults: the african-predict study. Hypertens Res. (2021) 44:840–49. 10.1038/s41440-021-00627-z 33564179

[25] Woodiwiss AJ Molebatsi N Maseko MJ Libhaber E Libhaber C Majane OH. Nurse-recorded auscultatory blood pressure at a single visit predicts target organ changes as well as ambulatory blood pressure. J Hypertens. (2009) 27:287–97. 10.1097/HJH.0b013e328317a78f 19155786

[26] de la Sierra A Pareja J Fernandez-Llama P Armario P Yun S Acosta E. Twenty-four-hour central blood pressure is not better associated with hypertensive target organ damage than 24-h peripheral blood pressure. J Hypertens. (2017) 35:2000–05. 10.1097/HJH.0000000000001431 28594710

[27] Andrikou E Tsioufis C Dimitriadis K Flessas D Chatzistamatiou V Grassos C. Parallel deterioration of albuminuria, arterial stiffness and left ventricular mass in essential hypertension: integrating target organ damage. Nephron Clin Pract. (2011) 119:c27–34. 10.1159/000324215 21654180

